# Heart Rate Variability, Insulin Resistance, and Insulin Sensitivity in Japanese Adults: The Toon Health Study

**DOI:** 10.2188/jea.JE20140254

**Published:** 2015-09-05

**Authors:** Isao Saito, Shinichi Hitsumoto, Koutatsu Maruyama, Wataru Nishida, Eri Eguchi, Tadahiro Kato, Ryoichi Kawamura, Yasunori Takata, Hiroshi Onuma, Haruhiko Osawa, Takeshi Tanigawa

**Affiliations:** 1 Department of Community Health Systems Nursing, Ehime University Graduate School of Medicine, Toon, Ehime, Japan; 2 Department of Total Medical Support Center, Ehime University Hospital, Toon, Ehime, Japan; 3 Department of Basic Medical Research and Education, University Graduate School of Medicine, Toon, Ehime, Japan; 4 Department of Molecular and Genetic Medicine, Ehime University Graduate School of Medicine, Toon, Ehime, Japan; 5 Department of Public Health, Okayama University Graduate School of Medicine, Dentistry and Pharmaceutical Sciences, Okayama, Japan; 6 Center for Education and Educational Research, Faculty of Education, Ehime University, Matsuyama, Japan; 7 Department of Public Health, Juntendo University Graduate School of Medicine, Tokyo, Japan

**Keywords:** heart rate variability, glucose intolerance, insulin sensitivity, epidemiology

## Abstract

**Background:**

Although impaired cardiac autonomic function is associated with an increased risk of type 2 diabetes in Caucasians, evidence in Asian populations with a lower body mass index is limited.

**Methods:**

Between 2009–2012, the Toon Health Study recruited 1899 individuals aged 30–79 years who were not taking medication for diabetes. A 75-g oral glucose tolerance test was used to diagnose type 2 diabetes, and fasting and 2-h-postload glucose and insulin concentrations were measured. We assessed the homeostasis model assessment index for insulin resistance (HOMA-IR) and Gutt’s insulin sensitivity index (ISI). Pulse was recorded for 5 min, and time-domain heart rate variability (HRV) indices were calculated: the standard deviation of normal-to-normal intervals (SDNN) and the root mean square of successive difference (RMSSD). Power spectral analysis provided frequency domain measures of HRV: high frequency (HF) power, low frequency (LF) power, and the LF:HF ratio.

**Results:**

Multivariate-adjusted logistic regression models showed decreased SDNN, RMSSD, and HF, and increased LF:HF ratio were associated significantly with increased HOMA-IR and decreased ISI. When stratified by overweight status, the association of RMSSD, HF, and LF:HF ratio with decreased ISI was also apparent in non-overweight individuals. The interaction between LF:HF ratio and decreased ISI in overweight individuals was significant, with the odds ratio for decreased ISI in the highest quartile of LF:HF ratio in non-overweight individuals being 2.09 (95% confidence interval, 1.41–3.10).

**Conclusions:**

Reduced HRV was associated with insulin resistance and lower insulin sensitivity. Decreased ISI was linked with parasympathetic dysfunction, primarily in non-overweight individuals.

## INTRODUCTION

The prevalence of type 2 diabetes is increasing rapidly worldwide, and fasting plasma glucose levels have risen globally since 1980.^1^ A recent review indicated that ethnic differences in insulin sensitivity were implicated in the pathogenesis of diabetes.^2^ Indeed, the prevalence of type 2 diabetes and the population attributable fraction for cardiovascular disease in Japan were estimated to be markedly increased, despite a low body mass index (BMI).^3^

Prospective epidemiological studies have reported that reduced heart rate variability (HRV) was associated with an increased risk of diabetes,^4^^,^^5^ hypertension,^6^ and cardiovascular disease.^7^^,^^8^ HRV is regulated by the combined activity of the sympathetic and parasympathetic nervous systems and is assessed by the beat-to-beat regulation of the heart rate.^9^ Although autonomic dysfunction is frequently recognized in patients with advanced diabetes as a form of autonomic neuropathy, it has been hypothesized that the action of insulin itself may be related to autonomic dysfunction in the early stage of glucose intolerance.^10^^–^^14^ In addition, autonomic dysfunction is associated closely with physical activity,^15^^,^^16^ behavioral factors,^17^ and psychosocial factors, such as social hierarchy,^18^ which can be explained in part by the hypothalamic-pituitary-adrenal axis.

The 5-min normal-to-normal (RR) interval measurement for assessment of cardiac autonomic control has been recommended as the standard method for both time and frequency domain analysis.^9^ The ARIC study demonstrated that decreased standard deviation of the normal-to-normal interval (SDNN), measured using RR intervals recorded for 2.5 min, increased the risk of cardiovascular disease and new-onset type 2 diabetes in the general population.^4^^,^^5^

Although autonomic dysfunction predicts events in Caucasians, it remains to be determined if a lower HRV is associated with post-load plasma blood glucose, insulin concentrations, and insulin resistance and sensitivity. This applies particularly to Asian populations, which tend to have low body weight and a genetic background for reduced insulin sensitivity compared to Caucasians.^2^ We therefore conducted a cross-sectional study on the association between HRV and type 2 diabetes in the general Japanese population. We used a 75-g oral glucose tolerance test (OGTT) and fasting blood glucose and insulin concentrations to diagnose type 2 diabetes and assess insulin resistance and sensitivity.

## METHODS

### Study subjects

From 2009 to 2012, the Toon Health Study (THS) recruited 2030 men and women who were 30–79 years of age. Only subjects who were not taking medication for diabetes and who did not show atrial fibrillation on an electrocardiogram (ECG) were included. Individuals who did not undergo a 75-g OGTT due to gastrectomy or who had a high fasting blood glucose (≥7.8 mmol/L) measured by self-monitoring of blood glucose were excluded. Using these criteria, 1899 individuals were included in the analysis.

The THS study was designed as a longitudinal epidemiological study for residents living in Toon City, Ehime Prefecture, Japan.^19^ Toon City is located on Shikoku Island in a rural area of the southern part of Japan and has a population of approximately 35 000. The goal of the THS is to identify novel environmental and genetic risk factors related to cardiovascular disease and type 2 diabetes.

Written informed consent was obtained from all the participants. The study protocol was approved by the Human Ethics Review Committees of Ehime University Graduate School of Medicine.

### Measurements

Overnight fasting blood samples were drawn from the antecubital vein into vacuum tubes containing a serum separator gel (for glucose and blood chemistry). The serum tube was centrifuged immediately at 3000 × g for 15 min, and the separated serum was sent to the laboratory for analysis. BMI was calculated as weight divided by height squared. Overweight was defined as a BMI ≥25 kg/m^2^.

### Blood examinations

Enzymatic methods were used to measure serum total cholesterol and triglyceride levels. Low-density lipoprotein cholesterol and high-density lipoprotein cholesterol were measured using the direct homogeneous method. The lipid measurements were standardized using the Center for Disease Control National Heart Lung and Blood Institute’s Lipid Standardization Program.^20^ Serum glucose was measured by the hexokinase method (Sysmex, Kobe, Japan) using an automatic analyzer (7600-D; Hitachi Co., Tokyo, Japan). Insulin was measured using the electrochemiluminescence method in ECLusys (Roche Diagnostics, Tokyo, Japan).

Blood pressure was measured twice in the sitting position after a rest of at least 5 min using an automatic sphygmomanometer (BP-103iII; OMRON Colin Co., Tokyo, Japan). The mean of the two measurements was used for analysis. Hypertension was defined as a systolic blood pressure ≥140 mm Hg or a diastolic blood pressure (DBP) ≥90 mm Hg or the current use of any antihypertensive medication.

### 75-g OGTT, HOMA-IR, and ISI assessments

All participants underwent an OGTT after at least a 10-h fast, and 1-h- and 2-h-postload glucose and insulin concentrations were measured by standard laboratory methods. The American Diabetes Association criteria for fasting and 2-h-postload glucose levels were used to identify normal or impaired glucose tolerance and type 2 diabetes.^21^ Impaired glucose tolerance (IGT) was defined as a 2-h-postload glucose level of 7.8–11.1 mmol/L, and impaired fasting glucose was defined as a fasting glucose level of 5.6–7.0 mmol/L. The homeostasis model assessment index for insulin resistance (HOMA-IR) was calculated as fasting insulin [µU/mL] × fasting glucose [mg/dL]/405.^22^ The insulin sensitivity index (ISI) was calculated using Gutt’s equation^23^ as: m/[mean glucose]/log[mean insulin], where m = [75 000 + (0 min glucose − 120 min glucose) × 0.19 × body weight (kg)]/120.

### Lifestyle

A self-administrated questionnaire was used to assess medical history, smoking habit, and alcohol consumption. The amount of ethanol consumed per week was evaluated by measuring the weekly frequency of drinking and the type of alcoholic beverage consumed (beer, sake, whiskey, *shochu* [a distilled liquor], or wine). A regular drinker was defined as alcohol consumption ≥1 g/week. Physical activity levels were assessed using a validated questionnaire, which consisted of 14 questions on occupation, locomotion, housework, sleep time, and leisure time physical activities. The responses for each physical activity were converted to metabolic equivalents (METs), according to the compendium by Ainsworth et al, and expressed as METs·h/day.^24^

### Assessment of autonomic function

Analysis of HRV was used as a non-invasive tool to assess cardiac autonomic control of the heart (TAS9; YKC Co. Ltd, Tokyo, Japan). The pulse rate was recorded for 5 min using a fingertip pulse wave sensor, and the following time-domain measures of HRV were then determined: SDNN and square root of the mean squared differences of successive RR intervals (RMSSD). Power spectral analysis of the 5-min ECG recordings was used to obtain frequency-domain measures of HRV, and the power spectrum was then decomposed into its frequency components and quantified in terms of the relative intensity (power) of each component. The power spectrum was divided into four major frequency bands: high frequency (HF) (0.15–0.40 Hz), low frequency (LF) (0.04–0.15 Hz), very-low frequency (VLF) (0.003–0.04 Hz), and ultra-low frequency (ULF) (<0.003 Hz). The HF and LF power and the LF:HF ratio were used for further analysis.

To assess the reliability of the HRV parameters, the parameters were measured twice in each individual by the same method at an interval of 2 months (*n* = 37). The Spearman’s correlation coefficients of these parameters ranged from 0.24 to 0.68.

### Statistical analysis

Because of skewed distributions, SDNN, RMSSD, LF, and HF were log-transformed before analysis. The LF:HF ratio was calculated using the log-transformed LF and HF values. Differences in these HRV parameters between men and women were analyzed using *t*-tests. Triglyceride, fasting glucose, fasting insulin, HOMA-IR, and Gutt’s ISI values were log-transformed and expressed as geometric means and standard deviations and grouped according to sex. Differences between sexes were examined using the chi-square test. Sex- and age-adjusted means were computed by analysis of covariance. Odds ratios (ORs) and 95% confidence intervals (CIs) for the quartile of HRV-related parameters were calculated using logistic regression analysis adjusting for sex, age, BMI, use of antihypertensive agents, DBP, physical activity, smoking, and alcohol drinking. A test for linear trends was also performed using log-transformed values of SDNN, RMSSD, LF, HF, and LF:HF ratio as continuous variables. Statistical significance was assumed at *P* < 0.05. All statistical analyses were performed using SAS software, version 9.4 (SAS Institute, Inc., Cary, NC, USA).

## RESULTS

Table 1 shows the characteristics of the subjects grouped according to sex. The mean age was 57.5 years, the percentage of men was 34.3%, and mean BMI was 23.1 kg/m^2^. Although the glucose and insulin profiles of men were worse than those in women, the means of HRV parameters were not markedly different between the sexes, except for LF and the LF:HF ratio.

**Table 1.** Population characteristics in the Toon Health Study

	Men	Women	Total
Number	652	1247	1899
Age, years	58.5 (12.8)*	57.0 (12.5)	57.5 (12.6)
Body mass index, kg/m^2^	24.0 (3.0)***	22.6 (3.2)	23.1 (3.2)
Waist circumference, cm	85.8 (8.2)***	81.8 (9.2)	83.2 (9.1)
Systolic blood pressure, mm Hg	128.8 (18.1)***	124.1 (20.5)	125.7 (19.9)
Diastolic blood pressure, mm Hg	80.2 (11.3)***	73.8 (11.6)	76.0 (11.9)
Triglycerides^a^, mmol/L	1.22 (0.02)***	0.97 (0.02)	1.05 (0.02)
LDL-cholesterol, mmol/L	3.06 (0.77)	3.13 (0.77)	3.11 (0.77)
HDL-cholesterol, mmol/L	1.41 (0.35)***	1.66 (0.36)	1.58 (0.37)
Total cholesterol, mmol/L	5.14 (0.83)***	5.42 (0.86)	5.32 (0.86)
Fasting glucose^a^, mmol/L	5.29 (0.06)***	5.02 (0.06)	5.11 (0.06)
Fasting insulin^a^, mmol/L	35.4 (12.9)***	33.1 (24.0)	33.9 (33.4)
HOMA-IR^a^	1.20 (1.91)***	1.06 (1.77)	1.11 (1.83)
Gutt’s ISI^a^	1.82 (1.42)**	1.90 (1.38)	1.87 (1.39)
Medication for hypertension, %	24.9***	17.7	20.2
Medication for dyslipidemia, %	8.1***	15.6	13.1
Current smoker, %	18.6***	3.7	8.8
Regular drinker, %	74.7***	39.7	51.7
Physical activity, METs·h/day	34.9 (4.9)***	36.0 (4.3)	35.6 (4.5)
lnSNDD	3.66 (0.49)	3.62 (0.48)	3.64 (0.48)
lnRMSSD	3.34 (0.62)	3.35 (0.62)	3.35 (0.62)
lnLF	5.15 (1.26)***	4.92 (1.26)	5.00 (1.26)
lnHF	4.66 (1.26)	4.75 (1.22)	4.72 (1.24)
LF:HF ratio	1.13 (0.24)***	1.06 (0.22)	1.08 (0.23)

HDL, high-density lipoprotein; HF, high frequency; HOMA-IR, homeostasis model assessment index for insulin resistance; ISI, insulin sensitivity index; LDL, low-density lipoprotein; LF, low frequency; METs, metabolic equivalents; RMSSD, root mean square of successive difference; SDNN, standard deviation of normal-to-normal intervals.

*P < 0.05, **P < 0.01, ***P < 0.001 for difference vs women.

^a^Represented as geometric means and standard deviations.

Table 2 shows the sex- and age-adjusted means grouped by SDNN quartiles. The higher quartiles of SDNN had lower values of BMI, waist circumference, DBP, fasting glucose, fasting insulin, and HOMA-IR and Gutt’s ISI indices. The other HRV parameters showed similar associations (eTables 1–4).

**Table 2.** Sex- and age-adjusted means^a^ grouped according to quartiles of SDNN (*n* = 1899)

	Quartile of SDNN				*P* for difference

	Q1 (Low)	Q2	Q3	Q4 (High)	
Age, years	63.7	58.1	54.4	53.7	<0.001
Sex, % men	33.4	32.6	32.8	38.5	0.17
Body mass index, kg/m^2^	23.4	23.1	22.9	22.8	0.025
Waist circumference, cm	84.3	83.5	82.3	82.7	0.004
Systolic blood pressure, mm Hg	127.0	125.7	125.0	125.3	0.38
Diastolic blood pressure, mm Hg	77.3	76.6	75.6	74.5	0.001
Triglycerides^a^, mmol/L	1.07	1.09	1.02	1.02	0.062
LDL-cholesterol, mmol/L	3.09	3.18	3.09	3.06	0.11
HDL-cholesterol, mmol/L	1.56	1.57	1.57	1.60	0.26
Total cholesterol, mmol/L	5.31	5.39	5.28	5.31	0.23
Fasting glucose^a^, mmol/L	5.17	5.11	5.08	5.09	0.043
Fasting insulin^a^, mmol/L	36.0	34.5	32.7	32.4	0.019
HOMA-IR^a^	1.19	1.13	1.06	1.05	0.009
Gutt’s ISI^a^	1.76	1.88	1.89	1.95	<0.001
Medication for hypertension, %	21.7	20.0	18.1	20.9	0.50
Medication for dyslipidemia, %	15.2	11.8	12.2	13.0	0.39
Current smoker, %	9.9	9.8	8.7	6.9	0.32
Regular drinker, %	51.8	48.9	52.6	53.6	0.44
Physical activity, METs·h/day	35.3	35.7	35.7	35.9	0.27

HDL, high-density lipoprotein; HOMA-IR, homeostasis model assessment index for insulin resistance; ISI, insulin sensitivity index; LDL, low-density lipoprotein; METs, metabolic equivalents; SDNN, standard deviation of normal-to-normal intervals.

Values are adjusted for sex and age by analysis of covariance. Age and sex values are shown as crude means and percentages.

^a^Represented as geometric means.

**Figure 1.**
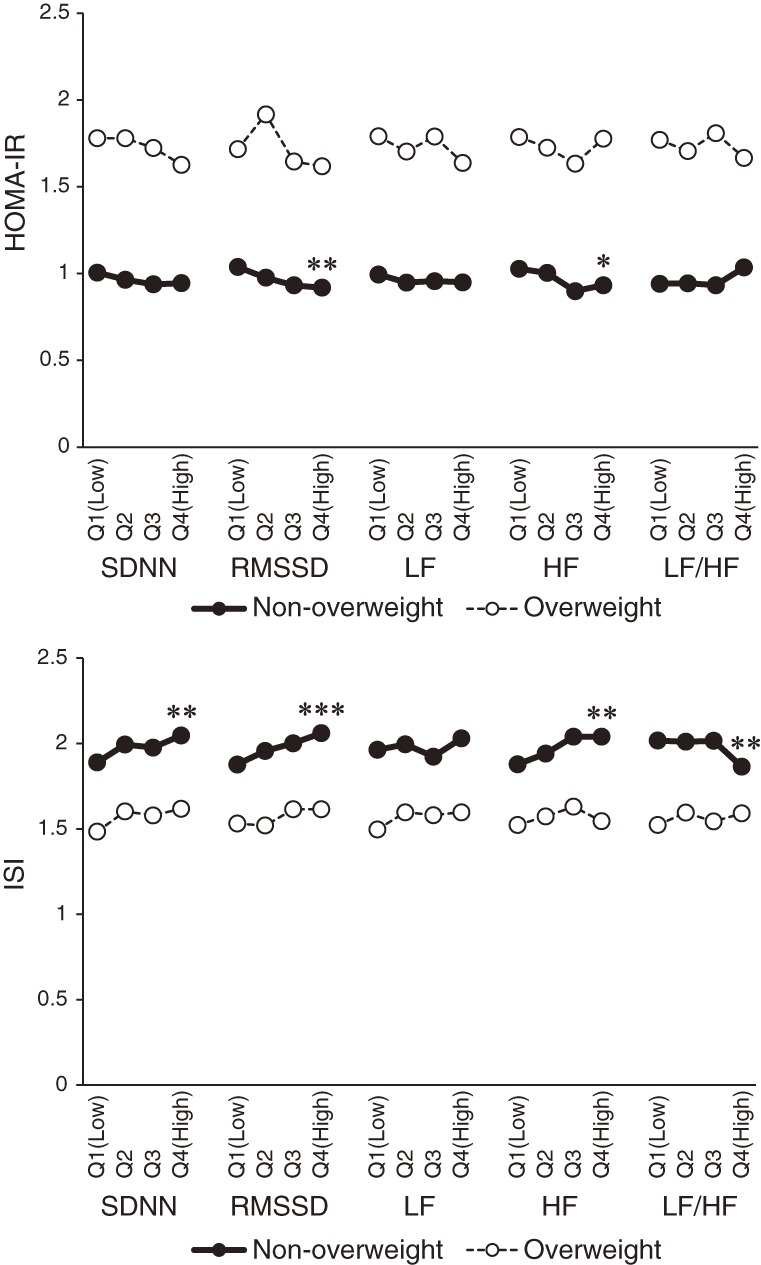
Sex- and age-adjusted means of the homeostasis model assessment index for insulin resistance (HOMA-IR) and insulin sensitivity index (ISI), grouped according to quartiles of heart rate variability parameters in both overweight and non-overweight subjects. The *P* values are for linear trends using continuous variables for each parameter in the model. *P < 0.05, **P < 0.01, ***P < 0.001.

The sex- and age-adjusted means of HOMA-IR and ISI grouped by HRV parameter quartiles are shown in Figure 1 for both overweight and non-overweight subjects. Significant associations of RMSSD and HF with HOMA-IR, SDNN, RMSSD, HF, and between LF:HF and ISI were observed in non-overweight individuals (BMI <25 kg/m^2^). There were no significant interactions of these parameters in overweight individuals.

Sex- and age-adjusted and multivariable-adjusted logistic regression analyses were performed after stratifying the HRV parameters and HOMA-IR and ISI data into quartiles. Table 3 shows the ORs for elevated HOMA-IR (highest quartile) or lower ISI (lowest quartile) grouped according to HRV parameter quartiles. The sex- and age-adjusted ORs for elevated HOMA-IR decreased significantly from the lowest to highest quartile of SDNN, RMSSD, LF, and HF. Although these associations were attenuated by adjustment for several confounders, including BMI (Model 2), RMSSD and HF remained significant (*P* for trend = 0.008 and 0.018, respectively). An increase in SDNN, RMSSD, and HF was also associated closely with lower ISI in both models. In contrast to changes in these three HRV parameters, an increase in LF:HF ratio was associated with an increased risk of lowered ISI. The multivariable-adjusted OR for decreased ISI was increased to 1.44 (95% CI, 1.05–1.98) in the highest quartile of LF:HF ratio (Model 2). We also investigated the sex-specific effect of HRV parameters on insulin but found no significant interactions (data not shown).

**Table 3.** Multivariable-adjusted odds ratios for elevated HOMA-IR (highest quartile) or lowered ISI (lowest quartile), grouped according to quartiles of heart rate variability parameters (*n* = 1899)

		HOMA-IR				Gutt’s ISI			
	
		Model 1		Model 2		Model 1		Model 2	
			
		OR	95% CI	OR	95% CI	OR	95% CI	OR	95% CI
SDNN	Q1 (lowest)	1.00		1.00		1.00		1.00	
	Q2	0.83	0.62–1.11	0.92	0.66–1.28	0.70	0.52–0.93	0.73	0.53–0.99
	Q3	0.77	0.57–1.04	1.01	0.72–1.42	0.67	0.49–0.90	0.79	0.57–1.09
	Q4 (highest)	0.65	0.47–0.88	0.79	0.56–1.13	0.53	0.39–0.72	0.59	0.42–0.82
	*P* for trend	0.002		0.062		<0.001		0.001	
RMSSD	Q1 (lowest)	1.00		1.00		1.00		1.00	
	Q2	0.83	0.63–1.11	0.80	0.60–1.07	0.75	0.57–1.00	0.83	0.61–1.12
	Q3	0.66	0.49–0.89	0.64	0.48–0.87	0.52	0.38–0.71	0.57	0.41–0.79
	Q4 (highest)	0.56	0.41–0.76	0.64	0.47–0.87	0.48	0.36–0.66	0.58	0.42–0.80
	*P* for trend	<0.001		0.008		<0.001		<0.001	
LF	Q1 (lowest)	1.00		1.00		1.00		1.00	
	Q2	0.76	0.57–1.02	0.74	0.53–1.04	0.81	0.60–1.08	0.84	0.61–1.15
	Q3	0.74	0.55–1.01	0.86	0.61–1.22	0.84	0.62–1.13	0.94	0.68–1.30
	Q4 (highest)	0.61	0.45–0.84	0.71	0.49–1.02	0.62	0.45–0.85	0.70	0.50–0.98
	*P* for trend	0.004		0.091		0.012		0.112	
HF	Q1 (lowest)	1.00		1.00		1.00		1.00	
	Q2	0.68	0.51–0.91	0.86	0.62–1.19	0.68	0.51–0.91	0.81	0.60–1.09
	Q3	0.57	0.42–0.78	0.77	0.54–1.08	0.49	0.36–0.68	0.60	0.43–0.84
	Q4 (highest)	0.57	0.42–0.77	0.73	0.52–1.04	0.50	0.37–0.68	0.60	0.43–0.83
	*P* for trend	<0.001		0.018		<0.001		<0.001	
LF:HF	Q1 (lowest)	1.00		1.00		1.00		1.00	
	Q2	0.90	0.67–1.23	0.86	0.61–1.22	0.88	0.64–1.20	0.86	0.62–1.20
	Q3	0.98	0.72–1.32	0.94	0.67–1.32	1.03	0.76–1.40	1.02	0.73–1.42
	Q4 (highest)	1.18	0.88–1.58	1.05	0.75–1.47	1.52	1.13–2.04	1.44	1.05–1.98
	*P* for trend	0.122		0.444		<0.001		0.001	

CI, confidence interval; HF, high frequency; HOMA-IR, homeostasis model assessment index for insulin resistance; ISI, insulin sensitivity index; LF, low frequency; OR, odds ratio; SDNN, standard deviation of normal-to-normal intervals; RMSSD, root mean square of successive difference.

Odds ratios and *P* values for tend were calculated after adjusting for sex and age in Model 1. In Model 2, they were adjusted further for body mass index, use of antihypertensive agents, diastolic blood pressure, physical activity, smoking and alcohol drinking.

**Figure 2.**
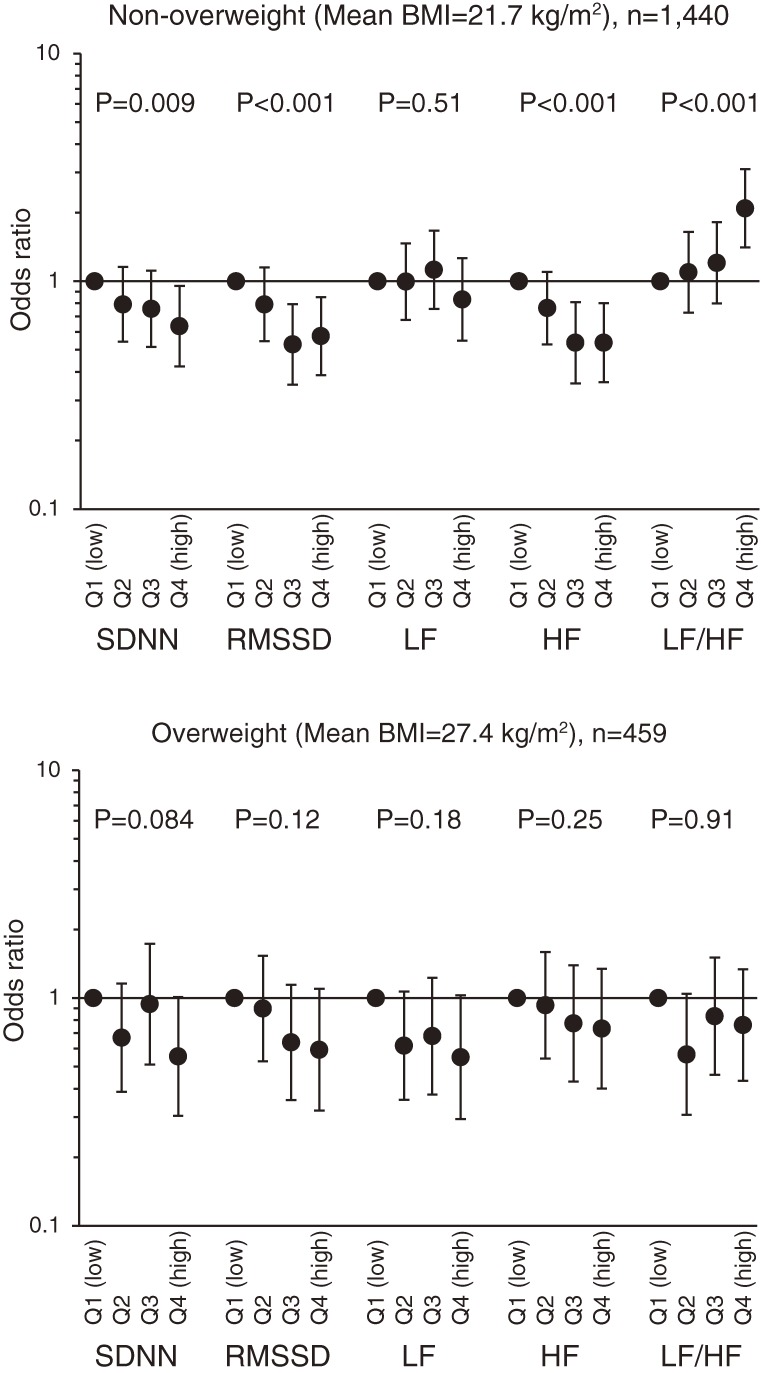
Multivariable-adjusted ORs for the lowest quartile of insulin sensitivity index (ISI), grouped according to quartiles of heart rate variability parameters in both overweight and non-overweight subjects. The ORs were adjusted for sex, age, BMI, use of antihypertensive agents, DBP, physical activity, smoking habit, and alcohol consumption. *P* values present the linear trends using continuous variables for each parameter in the model.

A separate logistic regression analysis was performed after dividing the subjects into overweight and non-overweight categories. This analysis showed that the associations of RMSSD, HF, and LF:HF ratio with lowered ISI were apparent in non-overweight individuals (mean BMI, 21.7 kg/m^2^) (Figure 2). The OR for lowered ISI in non-overweight subjects in the highest quartile of LF:HF ratio was 2.09 (95% CI, 1.41–3.10).

Table 4 shows the ORs of HRV parameters for the presence of IGT, IFG, or type 2 diabetes compared with normal glucose tolerance. SDNN, RMSSD, HF, and LF:HF ratio were associated with IGT and IFG as well as decreased ISI. For SNDD, RMSSD, and HR, the risk of IGT or IFG decreased from the lowest to highest quartile of all three HRV parameters. In contrast, the IGT or IFG risk increased from the lowest to highest quartile of LF:HF ratio. Type 2 diabetes was associated significantly with the quartile of SDNN and RMSSD, although there was no association with LF:HF ratio.

**Table 4.** Multivariable-adjusted odds ratios for IGT, IFG, or type 2 diabetes compared with NGT, grouped according to quartiles of the heart rate variability parameters

		IGT or IFG (*n* = 538) vs NGT (*n* = 1237)				Type 2 diabetes (*n* = 124) vs NGT (*n* = 1237)			
	
		Model 1		Model 2		Model 1		Model 2	
			
		OR	95% CI	OR	95% CI	OR	95% CI	OR	95% CI
SDNN	Q1 (lowest)	1.00		1.00		1.00		1.00	
	Q2	0.78	0.58–1.04	0.81	0.60–1.09	0.47	0.28–0.79	0.46	0.27–0.79
	Q3	0.84	0.62–1.14	0.96	0.70–1.30	0.54	0.32–0.93	0.60	0.35–1.04
	Q4 (highest)	0.59	0.43–0.80	0.66	0.48–0.90	0.46	0.27–0.79	0.47	0.27–0.81
	*P* for trend	0.001		0.012		0.003		0.004	
RMSSD	Q1 (lowest)	1.00		1.00		1.00		1.00	
	Q2	0.67	0.50–0.90	0.72	0.53–0.97	0.84	0.52–1.37	0.90	0.55–1.48
	Q3	0.54	0.40–0.73	0.60	0.44–0.81	0.51	0.29–0.89	0.52	0.30–0.92
	Q4 (highest)	0.57	0.42–0.77	0.66	0.49–0.90	0.45	0.26–0.79	0.48	0.27–0.85
	*P* for trend	<0.001		0.002		0.007		0.013	
LF	Q1 (lowest)	1.00		1.00		1.00		1.00	
	Q2	0.89	0.66–1.19	0.91	0.67–1.23	0.64	0.38–1.06	0.70	0.41–1.18
	Q3	0.93	0.68–1.26	1.00	0.73–1.38	0.76	0.45–1.27	0.81	0.48–1.38
	Q4 (highest)	0.83	0.61–1.13	0.92	0.67–1.27	0.59	0.33–1.03	0.61	0.34–1.08
	*P* for trend	0.343		0.833		0.098		0.126	
HF	Q1 (lowest)	1.00		1.00		1.00		1.00	
	Q2	0.72	0.54–0.96	0.81	0.60–1.09	0.88	0.55–1.42	1.00	0.62–1.64
	Q3	0.64	0.48–0.87	0.75	0.55–1.03	0.50	0.28–0.90	0.55	0.31–1.00
	Q4 (highest)	0.63	0.46–0.85	0.73	0.53–1.00	0.55	0.32–0.96	0.59	0.34–1.05
	*P* for trend	0.004		0.043		0.040		0.075	
LF:HF	Q1 (lowest)	1.00		1.00		1.00		1.00	
	Q2	0.81	0.59–1.11	0.80	0.58–1.11	1.21	0.73–2.01	1.14	0.68–1.91
	Q3	1.17	0.86–1.57	1.17	0.86–1.60	0.61	0.33–1.12	0.60	0.32–1.12
	Q4 (highest)	1.47	1.10–1.98	1.40	1.03–1.90	1.31	0.78–2.20	1.23	0.72–2.09
	*P* for trend	0.001		0.003		0.500		0.611	

CI, confidence interval; HF, high frequency; IGT, impaired glucose tolerance; IFG, impaired fasting glucose; LF, low frequency; NGT, normal glucose tolerance; OR, odds ratio; SDNN, standard deviation of normal-to-normal intervals; RMSSD, root mean square of successive difference.

Odds ratios and *P* values for tend were calculated after adjusting for sex and age in Model 1. In Model 2, they were adjusted further for body mass index, use of antihypertensive agents, diastolic blood pressure, physical activity, smoking, and alcohol drinking.

## DISCUSSION

This study showed that impaired cardiac autonomic function, evaluated by the HRV parameters of SDNN, RMSSD, HF, and LF:HF ratio, was associated with decreased ISI, even after adjustment for several confounders. SDNN is considered to be representative of both parasympathetic and sympathetic activities, whereas RMSSD and HF parameters mainly reflect parasympathetic activity. We suggest that parasympathetic inactivity, consequently leading to increased sympathetic activity, may reduce insulin sensitivity at the population level. The study also showed that reduced HRV was associated with lower ISI to a greater extent than either increased HOMA-IR or the presence of type 2 diabetes.

Of interest, decreased ISI was associated with decreased parasympathetic function, primarily in non-overweight individuals. Accordingly, the highest quartile of LF:HF ratio among non-overweight adults appeared to increase the ORs for decreased ISI. This suggests that insulin sensitivity may have been reduced by cardiac autonomic dysfunction more than by obesity in our subjects, despite obesity being considered a more important factor for the initial development of diabetes or metabolic syndrome.

In general, HOMA-IR reflects hepatic insulin sensitivity, whereas ISI represents both peripheral and hepatic insulin sensitivity, which have a higher correlation with the gold standard method for measuring insulin sensitivity: the euglycemic hyperinsulinemic clamp.^25^ The stronger associations of HRV with ISI than with HOMA-IR that we observed indicated that peripheral insulin sensitivity of skeletal muscles was markedly affected by impaired autonomic function.

Laitinen et al demonstrated that insulin infusion during a euglycemic clamp increased the LF:HF ratio and decreased the HF spectral component in individuals with insulin resistance (assessed by high C-peptide levels) but did not change the values in normal subjects with deficient insulin secretion capacity.^10^ These results suggest that cardiac autonomic dysfunction plays an important role in the progression of insulin resistance to type 2 diabetes. In the present study, the LF:HF ratio was associated with IGT/IFG, but not with diabetes. Although it is difficult to explain this discrepancy, we suspect that decreased HF may occur in an earlier state of glucose intolerance than LF. ORs for the highest quartile of SDNN and RMSSD were much lower in subjects with type 2 diabetes compared to subjects with normal glucose tolerance. Consequently, the LF:HF ratio might be attenuated under poor glucose conditions in conjunction with decreased whole autonomic function, including LF.

Decreased parasympathetic and increased sympathetic activities were associated strongly with changes in insulin sensitivity compared with indices of insulin resistance or glucose abnormalities in our study. Interestingly, a recent meta-analysis showed that, compared with Caucasians and Africans, East Asians (including Japanese) tended to have a high level of insulin sensitivity and a low level of insulin secretion, measured as the acute insulin response to glucose.^2^ Therefore, decreased insulin sensitivity may be a major contributor to the natural progression of insulin resistance to type 2 diabetes in East Asian people. Although it was difficult to identify whether the change in cardiac autonomic function occurred as a cause or result of diabetes or other physical conditions, several studies support the notion that sympathetic activation accompanied by decreased vagal tone may cause the progression of insulin resistance to diabetes. Cardiac autonomic dysfunction is associated with traditional cardiovascular disease (CVD) risk factors and is also linked to social determinants of health, such as CVD events and risk factors for CVD.^15^^,^^17^^,^^18^ Prospective epidemiological studies have also reported that reduced HRV is associated with an increased risk of diabetes^4^ and hypertension.^6^ On the basis of this evidence, our findings suggest that cardiac autonomic dysfunction, which reflects an imbalance of overall autonomic function, might be the underlying cause of an unfavorable CVD risk profile.^14^^,^^26^

Physiologically, the parasympathetic nervous system controls the direct secretion of insulin from beta cells, while the autonomic nervous system regulates glucose concentrations in the body. Diabetic autonomic neuropathy, which includes cardiac autonomic neuropathy, is a complication of type 1 and type 2 diabetes.^27^ Although clinical symptoms do not appear at the early stage of diabetes, the nerve damage occurs first in the vagus nerve, which then causes autonomic dysfunction related to the insulin secretion mechanism. Alternatively, insulin may have a modulating effect on autonomic tone,^12^ although induced hyperinsulinemia, stimulated by glucagon-like peptide-1 has been shown not to decrease vagal control.^28^ A recent review mentioned that inflammation activated by cytokines (such as IL-6), including adipocytokines in adipose tissue, potentially influenced the autonomic nervous system,^17^ leading to the conclusion that abdominal obesity may impair autonomic function. However, mean BMI was low in our population, and the association of HRV with insulin levels was observed in non-overweight individuals. Factors other than obesity, such as a sedentary lifestyle,^15^ high-fat food intake,^29^ or mental stress,^30^ may be key factors that partially influenced the autonomic imbalance linked with inflammation in the Japanese subjects in the current study. Alternatively, the hypothesis that the vagal anti-inflammatory pathway directly regulates inflammation may explain our results, especially for non-overweight individuals.^31^

Animal models suggest that the hepatic parasympathetic nerve dysfunction causes the development of skeletal muscle insulin resistance, linking with the actions of hepatic insulin sensitizing substance (HISS) released from the liver and of the glucose uptake in the skeletal muscle.^32^ The hepatic parasympathetic nerves regulate the HISS function; therefore, the impairment potentially lowers peripheral insulin sensitivity, which is consistent with our findings. This HISS hypothesis also implied that parasympathetic dysfunction precedes insulin resistance in the human body.^33^

Our study has several potential limitations. First, because of its cross-sectional design, we could not establish a causal relationship between decreased HRV and insulin resistance. IGT neuropathy, which is caused by autonomic nervous injury in peripheral fibers, may also have confounded this association.^34^ We did not assess the presence of autonomic neuropathy in the study. A future prospective longitudinal study is needed to determine if impaired cardiac autonomic function results in an increased risk of insulin resistance and development of diabetes. Second, although we adjusted for the use of agents related to cardiac autonomic function, such as antihypertensive drugs, in our statistical models, the effect of these agents could not be completely excluded. However, when we excluded patients taking antihypertensive drugs, the associations remained unchanged. Third, because the study participants were recruited voluntarily in the community, the data may not be representative of the general population in Japan.

The findings of this study support the hypothesis that autonomic dysfunction is a major factor in the development of decreased insulin sensitivity. As reported previously, this autonomic dysfunction is probably linked with environmental factors, such as a sedentary lifestyle and development of type 2 diabetes.^18^^,^^30^ We speculate that obesity itself may have a strong effect on the insulin response and may increase insulin levels regardless of HRV function.^35^ Therefore, the effect of HRV may become more evident in non-overweight individuals.

In conclusion, reduced HRV was associated with insulin resistance and lower insulin sensitivity. Decreased ISI was linked with parasympathetic dysfunction, primarily in non-overweight individuals.

## ONLINE ONLY MATERIALS

eTable 1. Sex- and age-adjusted means^a^ grouped according to quartiles of RMSSD (*n* = 1899).

eTable 2. Sex- and age-adjusted means^a^ grouped according to quartiles of LF (*n* = 1899).

eTable 3. Sex- and age-adjusted means^a^ b grouped according to quartiles of HF (*n* = 1899).

eTable 4. Sex- and age-adjusted means^a^ grouped according to quartiles of LF/HF ratio (*n* = 1899).

## References

[1] Danaei G, Finucane MM, Lu Y, Singh GM, Cowan MJ, Paciorek CJ, . National, regional, and global trends in fasting plasma glucose and diabetes prevalence since 1980: Systematic analysis of health examination surveys and epidemiological studies with 370 country-years and 2.7 million participants. Lancet. 2011;378:31–40. 10.1016/S0140-6736(11)60679-X 21705069

[2] Kodama K, Tojjar D, Yamada S, Toda K, Patel CJ, Butte AJ. Ethnic differences in the relationship between insulin sensitivity and insulin response: A systematic review and meta-analysis. Diabetes Care. 2013;36:1789–96. 10.2337/dc12-1235 23704681 PMC3661854

[3] Saito I. Epidemiological evidence of type 2 diabetes mellitus, metabolic syndrome, and cardiovascular disease in Japan. Circ J. 2012;76:1066–73. 10.1253/circj.CJ-11-1519 22453006

[4] Carnethon MR, Golden SH, Folsom AR, Haskell W, Liao D. Prospective investigation of autonomic nervous system function and the development of type 2 diabetes: The atherosclerosis risk in communities study, 1987–1998. Circulation. 2003;107:2190–5. 10.1161/01.CIR.0000066324.74807.95 12695289

[5] Liao D, Carnethon M, Evans GW, Cascio WE, Heiss G. Lower heart rate variability is associated with the development of coronary heart disease in individuals with diabetes: The atherosclerosis risk in communities (ARIC) study. Diabetes. 2002;51:3524–31. 10.2337/diabetes.51.12.3524 12453910

[6] Singh JP, Larson MG, Tsuji H, Evans JC, O’Donnell CJ, Levy D. Reduced heart rate variability and new-onset hypertension: Insights into pathogenesis of hypertension: The Framingham heart study. Hypertension. 1998;32:293–7. 10.1161/01.HYP.32.2.293 9719057

[7] Dekker JM, Crow RS, Folsom AR, Hannan PJ, Liao D, Swenne CA, . Low heart rate variability in a 2-minute rhythm strip predicts risk of coronary heart disease and mortality from several causes: The ARIC study. Atherosclerosis risk in communities. Circulation. 2000;102:1239–44. 10.1161/01.CIR.102.11.1239 10982537

[8] Gerritsen J, Dekker JM, TenVoorde BJ, Kostense PJ, Heine RJ, Bouter LM, . Impaired autonomic function is associated with increased mortality, especially in subjects with diabetes, hypertension, or a history of cardiovascular disease: The hoorn study. Diabetes Care. 2001;24:1793–8. 10.2337/diacare.24.10.1793 11574444

[9] Task Force of the European Society of Cardiology and the North American Society of Pacing and Electrophysiology. Heart rate variability. Standards of measurement, physiological interpretation, and clinical use. Task force of the european society of cardiology and the north american society of pacing and electrophysiology. Eur Heart J. 1996;17:354–81. 10.1093/oxfordjournals.eurheartj.a014868 8737210

[10] Laitinen T, Vauhkonen IK, Niskanen LK, Hartikainen JE, Länsimies EA, Uusitupa MI, . Power spectral analysis of heart rate variability during hyperinsulinemia in nondiabetic offspring of type 2 diabetic patients: Evidence for possible early autonomic dysfunction in insulin-resistant subjects. Diabetes. 1999;48:1295–9. 10.2337/diabetes.48.6.1295 10342819

[11] Festa A, D’Agostino R Jr, Hales CN, Mykkänen L, Haffner SM. Heart rate in relation to insulin sensitivity and insulin secretion in nondiabetic subjects. Diabetes Care. 2000;23:624–8. 10.2337/diacare.23.5.624 10834420

[12] Bergholm R, Westerbacka J, Vehkavaara S, Seppälä-Lindroos A, Goto T, Yki-Järvinen H. Insulin sensitivity regulates autonomic control of heart rate variation independent of body weight in normal subjects. J Clin Endocrinol Metab. 2001;86:1403–9. 11238539 10.1210/jcem.86.3.7307

[13] Schroeder EB, Chambless LE, Liao D, Prineas RJ, Evans GW, Rosamond WD, . Diabetes, glucose, insulin, and heart rate variability: The atherosclerosis risk in communities (ARIC) study. Diabetes Care. 2005;28:668–74. 10.2337/diacare.28.3.668 15735206

[14] Chang CJ, Yang YC, Lu FH, Lin TS, Chen JJ, Yeh TL, . Altered cardiac autonomic function may precede insulin resistance in metabolic syndrome. Am J Med. 2010;123:432–8. 10.1016/j.amjmed.2009.07.031 20399320

[15] Rennie KL, Hemingway H, Kumari M, Brunner E, Malik M, Marmot M. Effects of moderate and vigorous physical activity on heart rate variability in a british study of civil servants. Am J Epidemiol. 2003;158:135–43. 10.1093/aje/kwg120 12851226

[16] Soares-Miranda L, Sandercock G, Vale S, Santos R, Abreu S, Moreira C, . Metabolic syndrome, physical activity and cardiac autonomic function. Diabetes Metab Res Rev. 2012;28:363–9. 10.1002/dmrr.2281 22238216

[17] Thayer JF, Yamamoto SS, Brosschot JF. The relationship of autonomic imbalance, heart rate variability and cardiovascular disease risk factors. Int J Cardiol. 2010;141:122–31. 10.1016/j.ijcard.2009.09.543 19910061

[18] Hemingway H, Shipley M, Brunner E, Britton A, Malik M, Marmot M. Does autonomic function link social position to coronary risk? The whitehall ii study. Circulation. 2005;111:3071–7. 10.1161/CIRCULATIONAHA.104.497347 15939818

[19] Tabara Y, Saito I, Nishida W, Kohara K, Sakurai S, Kawamura R, . Relatively lower central aortic pressure in patients with impaired insulin sensitivity and resistance: The toon health study. J Hypertens. 2011;29:1948–54. 10.1097/HJH.0b013e32834abd06 21881525

[20] Nakamura M, Koyama I, Iso H, Sato S, Okazaki M, Kiyama M, . Measurement performance of reagent manufacturers by centers for disease control and prevention/cholesterol reference method laboratory network lipid standardization specified for metabolic syndrome-focused health checkups program in Japan. J Atheroscler Thromb. 2009;16:756–63. 10.5551/jat.1503 19763016

[21] The Expert Committee on the Diagnosis and Classification of Diabetes Mellitus. Report of the expert committee on the diagnosis and classification of diabetes mellitus. Diabetes Care. 1997;20:1183–97. 10.2337/diacare.20.7.1183 9203460

[22] Matthews DR, Hosker JP, Rudenski AS, Naylor BA, Treacher DF, Turner RC. Homeostasis model assessment: Insulin resistance and beta-cell function from fasting plasma glucose and insulin concentrations in man. Diabetologia. 1985;28:412–9. 10.1007/BF00280883 3899825

[23] Gutt M, Davis CL, Spitzer SB, Llabre MM, Kumar M, Czarnecki EM, . Validation of the insulin sensitivity index (ISI(0,120)): Comparison with other measures. Diabetes Res Clin Pract. 2000;47:177–84. 10.1016/S0168-8227(99)00116-3 10741566

[24] Ishikawa-Takata K, Naito Y, Tanaka S, Ebine N, Tabata I. Use of doubly labeled water to validate a physical activity questionnaire developed for the Japanese population. J Epidemiol. 2011;21:114–21. 10.2188/jea.JE20100079 21258166 PMC3899503

[25] Soonthornpun S, Setasuban W, Thamprasit A, Chayanunnukul W, Rattarasarn C, Geater A. Novel insulin sensitivity index derived from oral glucose tolerance test. J Clin Endocrinol Metab. 2003;88:1019–23. 10.1210/jc.2002-021127 12629079

[26] Flaa A, Aksnes TA, Kjeldsen SE, Eide I, Rostrup M. Increased sympathetic reactivity may predict insulin resistance: An 18-year follow-up study. Metabolism. 2008;57:1422–7. 10.1016/j.metabol.2008.05.012 18803948

[27] Vinik AI, Maser RE, Mitchell BD, Freeman R. Diabetic autonomic neuropathy. Diabetes Care. 2003;26:1553–79. 10.2337/diacare.26.5.1553 12716821

[28] Berkelaar M, Eekhoff EM, Simonis-Bik AM, Boomsma DI, Diamant M, Ijzerman RG, . Effects of induced hyperinsulinaemia with and without hyperglycaemia on measures of cardiac vagal control. Diabetologia. 2013;56:1436–43. 10.1007/s00125-013-2848-6 23404443

[29] Benthem L, Keizer K, Wiegman CH, de Boer SF, Strubbe JH, Steffens AB, . Excess portal venous long-chain fatty acids induce syndrome x via hpa axis and sympathetic activation. Am J Physiol Endocrinol Metab. 2000;279:E1286–93. 11093916 10.1152/ajpendo.2000.279.6.E1286

[30] Hamer M, Steptoe A. Association between physical fitness, parasympathetic control, and proinflammatory responses to mental stress. Psychosom Med. 2007;69:660–6. 10.1097/PSY.0b013e318148c4c0 17724255

[31] Cooper TM, McKinley PS, Seeman TE, Choo TH, Lee S, Sloan RP. Heart rate variability predicts levels of inflammatory markers: Evidence for the vagal anti-inflammatory pathway. Brain Behav Immun. 2014. doi:10.1016/j.bbi.2014.12.017. 10.1016/j.bbi.2014.12.017 25541185 PMC4476948

[32] Ribeiro RT, Lautt WW, Legare DJ, Macedo MP. Insulin resistance induced by sucrose feeding in rats is due to an impairment of the hepatic parasympathetic nerves. Diabetologia. 2005;48:976–83. 10.1007/s00125-005-1714-6 15830187 PMC2925889

[33] Lautt WW. A new paradigm for diabetes and obesity: The hepatic insulin sensitizing substance (HISS) hypothesis. J Pharmacol Sci. 2004;95:9–17. 10.1254/jphs.95.9 15153645

[34] Boulton AJ, Malik RA. Neuropathy of impaired glucose tolerance and its measurement. Diabetes Care. 2010;33:207–9. 10.2337/dc09-1728 20040677 PMC2797976

[35] Vinik AI, Maser RE, Ziegler D. Autonomic imbalance: Prophet of doom or scope for hope? Diabet Med. 2011;28:643–51. 10.1111/j.1464-5491.2010.03184.x 21569084 PMC3123705

