# Supplementary material for: Heart Rate Variability, Insulin Resistance, and Insulin Sensitivity in Japanese Adults: The Toon Health Study
Source: J Epidemiol. 2015 Sep 5;25(9):583–91. doi: 10.2188/jea.JE20140254 (PMC4549610; doi:10.2188/jea.JE20140254)
Supplement: eTable 1. [file je-25-583-s001.pdf]

eTable 1. Sex- and age-adjusted means[a] grouped according to quartiles of RMSSD (n=1,899)

	Quartile of RMSSD				
	Q1	Q2	Q3	Q4	*P* for difference
Age, years	62.1	57.8	54.5	55.9	<0.001
Men, %	35.2	35.7	31.6	34.9	0.53
Body mass index, kg/m^2	23.4	23.1	23.0	22.7	0.003
Waist circumference, cm	84.4	83.3	83.2	81.9	<0.001
Systolic blood pressure, mm Hg	127.8	126.2	124.4	124.6	0.013
Diastolic blood pressure, mm Hg	78.3	76.4	75.0	74.3	<0.001
Triglycerides[a], mmol/L	1.13	1.07	1.00	1.01	<0.001
LDL-cholesterol, mmol/L	3.12	3.15	3.07	3.09	0.43
HDL-cholesterol, mmol/L	1.55	1.56	1.58	1.61	0.023
Total cholesterol, mmol/L	5.33	5.34	5.28	5.33	0.68
Fasting glucose[a], mmol/L	5.15	5.15	5.08	5.07	0.010
Fasting insulin[a], mmol/L	36.5	35.3	32.8	31.3	<0.001
HOMA-IR[a]	1.20	1.17	1.07	1.02	<0.001
Gutt's ISI[a]	1.77	1.83	1.90	1.97	<0.001
Medication for hypertension, %	21.9	18.2	20.3	20.4	0.51
Medication for dyslipidemia, %	16.3	10.1	15.7	10.3	0.002
Current smoker, %	8.7	8.2	10.1	8.1	0.65
Regular drinker, %	50.9	52.0	52.0	51.9	0.98
Physical activity, METs·h/day	35.5	35.6	35.5	36.0	0.23

ISI, insulin sensitivity index; HDL, high-density lipoprotein; HOMA-IR, homeostasis model assessment index for insulin resistance; LDL, low-density lipoprotein; METs, metabolic equivalents; RMSSD, root mean square of successive difference.

Values are adjusted for sex and age by analysis of covariance. Age and sex values are shown in crude means and percentages.

[a] Represented as geometric means and standard deviations
