# Supplementary material for: Heart Rate Variability, Insulin Resistance, and Insulin Sensitivity in Japanese Adults: The Toon Health Study
Source: J Epidemiol. 2015 Sep 5;25(9):583–91. doi: 10.2188/jea.JE20140254 (PMC4549610; doi:10.2188/jea.JE20140254)
Supplement: eTable 2. [file je-25-583-s002.pdf]

eTable 2. Sex- and age-adjusted means[a] grouped according to quartiles of LF (n=1,899)

	Quartile of LF				
	Q1	Q2	Q3	Q4	*P* for difference
Age, years	64.2	58.5	54.4	52.9	<0.001
Men, %	28.1	31.3	36.8	41.1	<0.001
Body mass index, kg/m^2	23.2	23.3	22.9	22.8	0.10
Waist circumference, cm	83.8	83.5	82.9	82.5	0.12
Systolic blood pressure, mm Hg	126.2	125.3	126.1	125.3	0.79
Diastolic blood pressure, mm Hg	76.9	76.2	75.9	74.9	0.060
Triglycerides[a], mmol/L	1.06	1.07	1.03	1.04	0.68
LDL-cholesterol, mmol/L	3.11	3.11	3.12	3.09	0.95
HDL-cholesterol, mmol/L	1.57	1.58	1.58	1.58	0.94
Total cholesterol, mmol/L	5.32	5.34	5.32	5.30	0.93
Fasting glucose[a], mmol/L	5.14	5.11	5.09	5.11	0.56
Fasting insulin[a], mmol/L	35.8	33.8	33.7	32.2	0.055
HOMA-IR[a]	1.18	1.11	1.10	1.05	0.057
Gutt's ISI[a]	1.81	1.88	1.84	1.94	0.013
Medication for hypertension, %	23.9	16.6	20.0	20.2	0.035
Medication for dyslipidemia, %	14.2	13.6	13.0	11.4	0.62
Current smoker, %	11.8	9.5	6.6	7.3	0.028
Regular drinker, %	48.6	51.0	54.4	52.9	0.29
Physical activity, METs·h/day	35.5	35.6	35.6	35.8	0.81

ISI, insulin sensitivity index; HDL, high-density lipoprotein; HOMA-IR, homeostasis model assessment index for insulin resistance; LDL, low-density lipoprotein; LF, low frequency; METs, metabolic equivalents.

Values are adjusted for sex and age by analysis of covariance. Age and sex values are shown in crude means and percentages.

[a]Represented as geometric means and standard deviations
