# Supplementary material for: Heart Rate Variability, Insulin Resistance, and Insulin Sensitivity in Japanese Adults: The Toon Health Study
Source: J Epidemiol. 2015 Sep 5;25(9):583–91. doi: 10.2188/jea.JE20140254 (PMC4549610; doi:10.2188/jea.JE20140254)
Supplement: eTable 3. [file je-25-583-s003.pdf]

eTable 3. Sex- and age-adjusted means[a] b grouped according to quartiles of HF (n=1,899)

	Quartile of HF				
	Q1	Q2	Q3	Q4	*P* for difference
Age, years	62.7	58.6	54.0	54.6	<0.001
Men, %	37.8	36.4	31.0	32.1	0.081
Body mass index, kg/m^2	23.6	23.0	22.8	22.8	<0.001
Waist circumference, cm	84.9	83.1	82.5	82.2	<0.001
Systolic blood pressure, mm Hg	127.2	125.8	124.6	125.3	0.16
Diastolic blood pressure, mm Hg	78.1	76.3	75.0	74.5	<0.001
Triglycerides[a], mmol/L	1.12	1.06	1.01	1.01	0.004
LDL-cholesterol, mmol/L	3.13	3.16	3.05	3.08	0.12
HDL-cholesterol, mmol/L	1.56	1.55	1.59	1.60	0.17
Total cholesterol, mmol/L	5.36	5.36	5.27	5.30	0.29
Fasting glucose[a], mmol/L	5.17	5.12	5.06	5.10	0.010
Fasting insulin[a], mmol/L	37.0	34.9	31.7	32.1	<0.001
HOMA-IR[a]	1.23	1.14	1.03	1.05	<0.001
Gutt's ISI[a]	1.76	1.84	1.94	1.94	<0.001
Medication for hypertension, %	22.7	16.6	19.3	22.1	0.051
Medication for dyslipidemia, %	14.9	13.2	13.6	10.5	0.20
Current smoker, %	9.7	9.8	8.6	7.0	0.35
Regular drinker, %	52.6	50.2	52.0	52.1	0.86
Physical activity, METs·h/day	35.3	36.0	35.5	35.8	0.086

ISI, insulin sensitivity index; HDL, high-density lipoprotein; HF, high frequency; HOMA-IR, homeostasis model assessment index for insulin resistance; LDL, low-density lipoprotein; METs, metabolic equivalents.

Values are adjusted for sex and age by analysis of covariance. Age and sex values are shown in crude means and percentages.

[a] Represented as geometric means and standard deviations
