# Supplementary material for: Heart Rate Variability, Insulin Resistance, and Insulin Sensitivity in Japanese Adults: The Toon Health Study
Source: J Epidemiol. 2015 Sep 5;25(9):583–91. doi: 10.2188/jea.JE20140254 (PMC4549610; doi:10.2188/jea.JE20140254)
Supplement: eTable 4. [file je-25-583-s004.pdf]

eTable 4. Sex- and age-adjusted means[a] grouped according to quartiles of LF/HF ratio (n=1,899)

	Quartile of LF/HF ratio				
	Q1	Q2	Q3	Q4	*P* for difference
Age, years	60.4	57.2	55.7	56.4	<0.001
Men, %	24.9	29.5	38.2	44.9	<0.001
Body mass index, kg/m^2	22.8	23.0	23.2	23.3	0.049
Waist circumference, cm	82.6	82.6	83.3	84.2	0.020
Systolic blood pressure, mm Hg	125.4	125.4	125.2	126.9	0.38
Diastolic blood pressure, mm Hg	75.5	76.0	75.2	77.2	0.025
Triglycerides[a], mmol/L	1.04	1.02	1.05	1.09	0.15
LDL-cholesterol, mmol/L	3.06	3.08	3.12	3.16	0.19
HDL-cholesterol, mmol/L	1.59	1.58	1.57	1.57	0.77
Total cholesterol, mmol/L	5.29	5.29	5.33	5.38	0.32
Fasting glucose[a], mmol/L	5.09	5.12	5.08	5.16	0.071
Fasting insulin[a], mmol/L	33.6	32.7	33.4	35.7	0.095
HOMA-IR[a]	1.10	1.07	1.09	1.18	0.065
Gutt's ISI[a]	1.88	1.91	1.90	1.78	0.004
Medication for hypertension, %	22.6	21.1	19.6	17.3	0.18
Medication for dyslipidemia, %	13.0	11.1	13.6	14.6	0.43
Current smoker, %	8.5	9.4	8.6	8.7	0.96
Regular drinker, %	50.0	52.2	51.0	53.7	0.65
Physical activity, METs·h/day	35.7	35.4	35.8	35.6	0.65

ISI, insulin sensitivity index; HDL, high-density lipoprotein; HF, high frequency; HOMA-IR, homeostasis model assessment index for insulin resistance; LDL, low-density lipoprotein; LF, low frequency; METs, metabolic equivalents.

Values are adjusted for sex and age by analysis of covariance. Age and sex values are shown in crude means and percentages.

[a] Represented as geometric means and standard deviations
